# The iconic motivation for the morphophonological distinction between noun–verb pairs in American Sign Language does not reflect common human construals of objects and actions

**DOI:** 10.1017/langcog.2022.20

**Published:** 2022-10-26

**Authors:** Jennie E. Pyers, Karen Emmorey

**Affiliations:** 1 Wellesley College, Psychology Department, Wellesley, MA, USA; 2 San Diego State University, School of Speech, Language and Hearing Sciences, San Diego, CA, USA

**Keywords:** sign language, iconicity, lexical categories, cognitive biases, American Sign Language

## Abstract

Across sign languages, nouns can be derived from verbs through morphophonological changes in movement by (1) movement reduplication and size reduction or (2) size reduction alone. We asked whether these cross-linguistic similarities arise from cognitive biases in how humans construe objects and actions. We tested nonsigners’ sensitivity to differences in noun–verb pairs in American Sign Language (ASL) by asking MTurk workers to match images of actions and objects to videos of ASL noun–verb pairs. Experiment 1a’s match-to-sample paradigm revealed that nonsigners interpreted all signs, regardless of lexical class, as actions. The remaining experiments used a forced-matching procedure to avoid this bias. Counter our predictions, nonsigners associated reduplicated movement with actions not objects (inversing the sign language pattern) and exhibited a minimal bias to associate large movements with actions (as found in sign languages). Whether signs had pantomimic iconicity did not alter nonsigners’ judgments. We speculate that the morphophonological distinctions in noun–verb pairs observed in sign languages did not emerge as a result of cognitive biases, but rather as a result of the linguistic pressures of a growing lexicon and the use of space for verbal morphology. Such pressures may override an initial bias to map reduplicated movement to actions, but nevertheless reflect new iconic mappings shaped by linguistic and cognitive experiences.

## 1. Introduction

Iconicity, typically defined as the resemblance of a symbol to its referent, is an essential tool in the creation of language, spoken or signed (e.g., Imai & Kita, 2014; Perniss & Vigliocco, 2014). Humans readily generate novel iconic symbols in the laboratory, and modern-day languages bear traces of iconic origins (e.g., Blasi et al., 2016; Perlman et al., 2015; Taub, 2001). Iconicity in language can appear in the lexicon, grammar, and discourse of signed or spoken languages. Some iconic mappings reflect a correspondence between the linguistic form and common human cognitive construals of meaning. Here we ask whether the morphophonological distinctions between nouns and verbs in American Sign Language (ASL) are iconically driven by cognitive construals of objects and actions.

Languages around the world make systematic distinctions between nouns and verbs via a variety of methods, for example, inflection, reduplication, and in some cases, tone. In less inflectionally rich languages, such as English, syntax often disambiguates lexical class when the noun and verb are homophonous, as in *hug* (e.g., “Hug me!” vs. “Give me a hug.”). In some cases, when the segmental phonology of the noun and verb is identical, changes in prosody, specifically stress placement, provide information about the lexical class. In English, nouns in such pairs are articulated with the first syllable stressed and verbs with the second syllable stressed (e.g., PROgress vs. proGRESS).

Both within and across sign languages, nouns can be derived from verbs through a variety of methods, including changing mouthing (Johnston, 2001), combining multiple signs (Tkachman & Sandler, 2013), and changing the morphophonological structure of the verb, including modifying the handshape or movement of the sign (Abner, 2017, Kimmelman, Klezovich, & Moroz, 2018; Padden et al., 2015). Reduplication of the verb is a robust form of noun derivation in sign languages (Abner, 2017; Supalla & Newport, 1978). Accompanying the reduplication of a signed verb is a change in the quality of sign movement such that the movement of the noun is smaller and more restrained relative to the verb, leading to a shorter articulation time (Hunger, 2006). The contrastive use of reduplication and movement size is applied to only those verbs that have a single movement, typically perfective verbs which have a “clear spatial endpoint” and are noniterative, for example, FLY-BY-AIRPLANE, SIT (Supalla & Newport, 1978). Reduplication, however, cannot be the only means of noun derivation, as reduplicated movement can be applied to verbs in sign languages to convey additional semantic information such as event duration, telicity (whether or not an action is completed), and event frequency (Supalla & Newport, 1978). Imperfective verbs, for example, are expressed with a reduplicated large movement (e.g., PEDAL_A_BIKE, SWEEP), and their derived nouns are articulated with the same, but reduced, reduplicated movement. In these cases, reduplication iconically represents the iterative meaning of the verb and cannot provide contrastive information that distinguishes the noun and verb forms in the same way that it does for perfective verbs. Instead, the size of the movement is contrastive: the verbs are articulated with a large reduplicated movement, while the nouns are articulated with a smaller reduplicated movement (Supalla & Newport, 1978).

**Fig. 1.**
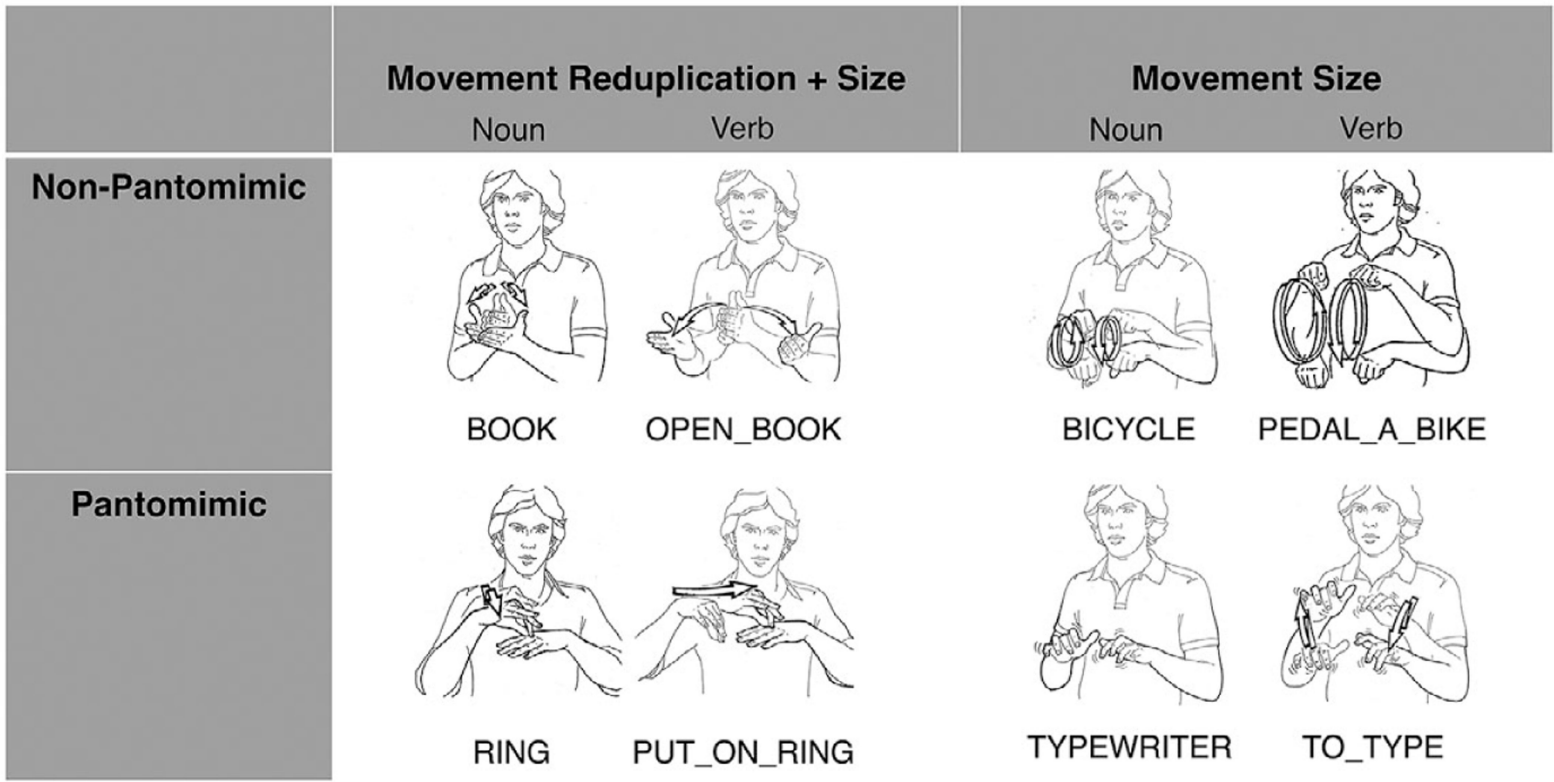
Examples of noun-verb pairs that differ as a function of either movement reduplication + size or movement size alone and that differ as a function of whether they are pantomimic or not. Illustrations created by Frank Allen Paul and collected by Ursula Bellugi at the Salk Institute.

In the cases where movement is modified to derive nouns from verbs in ASL, two general principles can be applied depending on the semantics of the verb: (1) *movement reduplication* + *size*, or (2) *movement size only*. In the first case, when the verb does not have a reduplicated movement, as in the ASL sign, OPEN_BOOK, the noun is derived by reduplication and a reduction in movement, yielding the ASL sign BOOK (Fig. 1). In the second case, reduplication is already present in the verb, as in the example PEDAL_A_BIKE, and cannot function as a contrastive morphophonological feature, so the reduction in movement size alone distinguishes the noun from the verb in the pair, as in the ASL example BICYCLE (Fig. 1). Note that reduplication is a feature of all nouns illustrated in Fig. 1, but it only serves as a contrastive feature in the noun–verb pairs BOOK/OPEN_BOOK and RING/PUT_ON_RING because the verbs in both cases do not have reduplicated movement.

If a common cognitive construal of actions and objects underlies the morphophonological contrasts that distinguish nouns from verbs, these contrasts may be seen in systematic iconic mappings between aspects of the phonological form and lexical class. Within a cognitive grammar approach to iconicity, Wilcox (2004) argues that the grammatical distinction between nouns and verbs is represented by different schemas in which nouns profile “a thing” in a region, while verbs profile “a process” distributed across time. Specifically, Wilcox (2004) suggests that the large articulated movement of a verb makes the movement of the sign form more salient and iconically emphasizes the process-oriented nature of actions. In contrast, restrained, reduplicated movements associated with nouns reflect object schemas because these noun forms “are articulated in a region of conceptual space occupied by *things*” (p. 132; italics in the original). Expanding on this idea, Lepic and Padden (2017) have highlighted that the contrast in movement repetition and size between paired nouns and verbs reflects a contrast in iconic meaning between objects and actions, with restricted, reduplicating movement deemphasizing action and thereby redirecting attention to the object being acted upon. Under the *structure mapping* and *analogue building* views of iconicity (Emmorey, 2014; Taub, 2001), phonological features of a sign are structurally aligned with semantic features of its referent, and this alignment must be structure preserving. For example, movement direction is a relational construct, and thus a sign with a single path movement is likely to align with a verb that encodes relational information (e.g., perfective verbs with an endpoint). With this proposed system of iconic mappings, we predict that nonsigners have a bias to see large movements and single path movements as actions because they emphasize the process-oriented nature of actions, and small reduplicated movements as objects because such movement focuses on a spatial region and deemphasizes action.

One or both of these derivational strategies appear, alongside others, across many different sign languages to varying degrees. While we observe expected similarities in the derivational strategies that leverage changes in the sign’s movement among related sign languages, such as those related to Old French Sign Language (LSF) including modern LSF (Moody, 1983 as cited in Vogel, 2005), Quebec Sign Language (LSQ; Bouchard et al.,2005), ASL (Abner etal., 2019; Supalla & Newport,1978), Italian Sign Language (LIS; Pizzuto & Corazza, 1996), and Russian Sign Language (RSL; Kimmelman, 2009), these noun–verb movement patterns appear in sign languages unrelated to LSF, such as those from the family of British Sign Language (BSL) including Australian Sign Language (AUSLAN; Johnston, 2001) and New Zealand Sign Language (NZSL; Collins-Ahlgren, 1990). These morphophonological patterns are observed even in relatively young sign languages like Israeli Sign Language (ISL; Tkachman & Sandler, 2013). Similarities across sign languages in marking morphophonological distinctions in noun–verb pairs by modifying a sign’s movement points to possible universals in structural iconicity that create these cross-linguistic similarities (e.g., Schlenker et al., 2013; Wilbur, 2003). Indeed, cross-linguistic similarity in morphophonological distinctions in verb telicity among unrelated sign languages has been documented (Strickland et al., 2015). Moreover, the phonological changes in movement that denote changes in telicity are readily recognized by nonsigners, indicating that the iconic mapping of movement taps an underlying cognitive construal of verbs that is universal to all humans learning language.

These cognitive construals of actions and objects, however, may compete with biases in interpreting other iconic mappings. Take the case of pantomimic iconicity which depicts how a human engages in an action or with an object. When signs have two variants, for example, a more pantomimic variant that shows how an instrument is handled (hand-as-hand iconicity) and a less pantomimic variant that shows the shape of the instrument in the handshape of the sign (hand-as-object iconicity), signers tend to prefer the pantomimic handling variant as a verb and the instrument variant as a noun (Brentari et al., 2013; Padden et al., 2015). Although nonsigners produce handling gestures for both actions and objects (Padden et al., 2015; van Nispen et al., 2017), they are more likely to use pantomimic gestures (e.g., handling gestures) than other types of gestures for actions, and do so more often than for objects (Ortega & Özyürek, 2019; Padden et al., 2015). In addition, nonsigners perceive handling signs as more iconic than deaf signers, although both groups perceive hand-as-hand iconicity as more iconic than hand-as-object iconicity (Sehyr & Emmorey, 2019). As such, a heightened perception of pantomimic signs as more iconic combined with a greater bias to treat handling gestures as action-oriented may compete with other construals of reduced movement size and/or reduplication as being associated with objects.

Across a set of experiments, we tested nonsigners’ sensitivity to two ways that nouns are derived from verbs: *movement reduplication* + *size* and *movement size only*. We hypothesized that these derivational strategies originate in universal human cognitive construals of actions and objects that can be iconically mapped to elements of the morphophonological structure of the sign. The clearest indicator of this construal would be the observation that nonsigners systematically associate large, nonreduplicated movements in signs with actions and small reduplicated movements with objects. We additionally hypothesized that pantomimic iconicity might affect nonsigners’ assignment of noun–verb signs to objects and actions respectively. Accordingly, we consider the following possible outcomes:

(1) Nonsigners will correctly map ASL verbs to actions and ASL nouns to objects regardless of morphological strategy.
(2a) Nonsigners will be sensitive to the *movement reduplication* + *size* rule but not to the *movement size only* rule because *movement reduplication* + *size* provides two iconic cues to support the mapping of verbs to actions and nouns to objects.
(2b) Alternatively, because both patterns include size reduction as a meaningful contrast between nouns and verbs, nonsigners may be sensitive to the *movement size* rule as opposed to reduplication.
(3) When exposed to both rules, as is the case with natural language, nonsigners may struggle to extract the relevant pattern to distinguish nouns from verbs.
(4) Nonsigners may not be able to distinguish nouns from verbs with pantomimic signs because they will interpret all pantomimic signs as depicting human actions.

We structured our experiments to test sensitivity to each rule separately and then to both rules at the same time. In addition, we tested nonsigners’ sensitivity to each rule with nonpantomimic and pantomimic signs separately. We speculated that by isolating each variable we would be better able to detect any effects among the nonsigners. In Experiment 1 we investigated nonsigners’ interpretation of the meaning of nonpantomimic nouns and verbs in ASL that differed according to the *movement reduplication* + *size* rule and the *movement size* rule, using first a match-to-sample paradigm (Experiment 1a) and then a forced-matching paradigm (Experiments 1b and 1c). We tested sensitivity to these distinctions using both a between subjects and within-subjects design. We included ASL signers in Experiments 1a and one condition of Experiment 1b to confirm that they were indeed sensitive to these linguistic distinctions in the way that we would expect. Finally, in Experiment 2 we used the same between- and within-subjects design to investigate these effects with pantomimic signs, as this type of iconicity may be especially salient for nonsigners.

## 2. General method

### 2.1. Item selection

We first identified a list of 104 concrete noun–verb pairs in ASL. Of these pairs, 72 followed the *movement reduplication* + *size* rule, where verbs are articulated with a single movement in a large signing space, and nouns are articulated with a reduplicated movement in a reduced signing space (Fig. 1). The remaining 32 followed the *movement size* rule, where both verbs and nouns are produced with reduplicated movement, but nouns are articulated in a smaller signing space relative to verbs. We coded whether signs were pantomimic or not (Fig. 1). We operationalized pantomimic signs as being handling signs or conventional pantomimes that involve manipulating objects (e.g., holding a Y handshape for TELEPHONE/TO_CALL). Fifty-eight of the pairs included pantomimic iconicity and 46 did not.

**Fig. 2.**
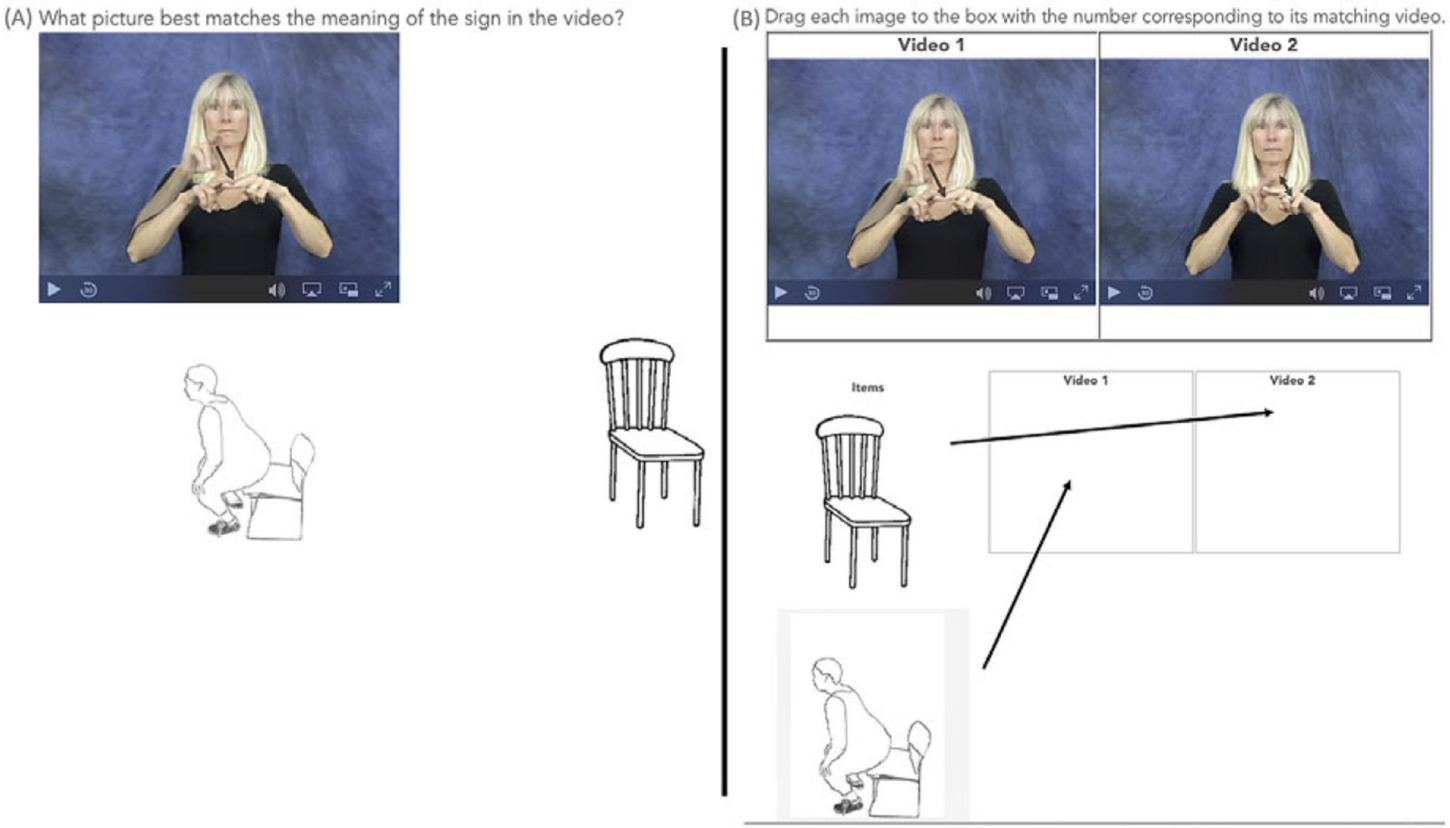
Images of the two methods of assessing sensitivity to the movements that distinguish nouns from verbs (A) match-to-sample (Experiment 1a) and (B) forced-matching (Experiments 1b-2c).The arrows in (B) denote the path from the image to the correct location below the video.

We then identified separate black and white line drawings of images that could depict either the object or the action associated with the object for each of the noun–verb pairs (see Fig. 2 for an example of images depicting a chair and sitting on a chair). Using 3 batches of pictures we asked Amazon Mechanical Turk (mTurk) master workers (Batch 1: *n* = 12, Batch 2: *n* = 15, Batch 3: *n* = 10) to rate individual line drawings on how object-like or action-like the image was, using a scale of 1 (object) to 7 (action). Workers were paid in accordance with US national minimum wage ($7.25/hour) and did not participate in any subsequent experiments reported in this paper. We selected pictures that were rated more object-like (average rating of 2 or lower) to pair with nouns and pictures that were rated more action-like (average rating of 6 or higher) to pair with verbs. We were able to identify a subset of 61 noun–verb pairs in ASL for which we had good line drawings to represent both the object and actions. We used items from this subset of noun–verb pairs for all experiments (see Appendix). The picture and video stimuli are available on OSF (https://osf.io/q3hjg/?view_only=55c01f66aace43dc89555297de1359fb).

### 2.2. Participants

For each experiment, we recruited nonsigners from the pool of Amazon MTurk workers who have been designated “master” by the MTurk system for their high successful and reliable completion rates. All recruited MTurk workers reported no experience with a sign language. None of the MTurk workers participated in more than one of the experiments. Informed consent was obtained from all participants in accordance with the Institutional Review Board at Wellesley College. Our target sample size for each between-subject condition was 40 for a total of 80 participants, and our target sample size for each within-subject experiment was 40.^1^

### 2.3. Statistics

All statistics were conducted using R version 4.0.3 (R Core Team, 2021). For each experiment, we ran a mixed effects logistic regression using the lme4 package (Bates et al., 2015) and the function “glmr()” to explicitly investigate the effects of rule type on whether nonsigners correctly detected the ASL-like mapping. We included participants and items as random intercepts and rule type as a fixed effect. For all within-subjects analyses, we included by-participant random slopes. For the match to sample paradigm of Experiment 1a, where we only examined sensitivity to one rule, *movement size* + *reduplication*, we included lexical class (noun/verb) instead of rule type as a fixed effect.

To explicitly test the effects of rule type (*movement reduplication* + *size* vs. *movement size only*), iconicity type (nonpantomimic vs. pantomimic), and the interaction between rule type and iconicity type, we combined the data from all Experiments in a single mixed-effect logistic regression, including participants and items as random intercepts and rule type, iconicity type, and the interaction between rule type and iconicity type as fixed effects. Because there were some within-subjects participants we included by-participant random slopes.

Across all analyses the fixed factor of rule type was sum coded as either *movement reduplication* + *size* (−1) or *movement size only* (+1), with *movement reduplication* + *size* as the reference category; the fixed factor of iconicity type was sum coded as either *nonpantomimic* (−1) or *-pantomimic* (+1) with nonpantomimic as the reference category. Data files and analysis code can be found at: https://osf.io/q3hjg/?view_only=55c01f66aace43dc89555297de1359fb.

## 3. Experiment 1: nonpantomimic signs

### 3.1. Experiment 1a: testing the sensitivity of the movement reduplication + size rule with a match-to-sample design

#### 3.1.1. Method

##### Participants.

Thirty-seven of the 40 recruited MTurk workers completed all trials and passed all checks. The final sample included 22 males and 15 females (27 White, 4 Black or African American, 3 Asian, 1 Hispanic or Latino, 1 mixed-race, and 1 no answer). We also recruited 14 ASL signers (4 M and 10 F; 9 native signers and 5 early signers) through personal networks and snowball sampling. The deaf ASL signers included 12 White, 1 Asian, and 1 American Indian. The deaf participants were entered into a lottery to win one of two $50 gift cards.

##### Design and procedure.

We selected 20 noun–verb pairs that had no pantomimic iconicity and that followed the *movement reduplication* + *size* rule for inclusion in the study (see Appendix). In a within-subjects, match-to-sample paradigm presented in Qualtrics, participants saw a randomized presentation of either the noun sign (*n* = 10) or the verb sign (*n* = 10) from the noun–verb minimal pair, alongside the line-drawn images of both the related object and action referent (Fig. 2a). Placement of the line drawings, was randomized across trials. Participants were instructed to select the picture that best illustrated the sign. We generated two counterbalanced orders of administration such that if the noun sign of the minimal pair was presented in one order, then its verb counterpart was presented in the other, and vice versa. Participants were randomly assigned to complete one of the two orders of administration (20 nonsigners and 7 signers completed one order, and 17 nonsigners and 7 signers completed the second order; the three nonsigners who did not complete all trials or pass the attention checks happened to be randomly assigned to the second order). It took participants on average 7 minutes to complete this study.

#### 3.1.2. Results and discussion

**Fig. 3.**
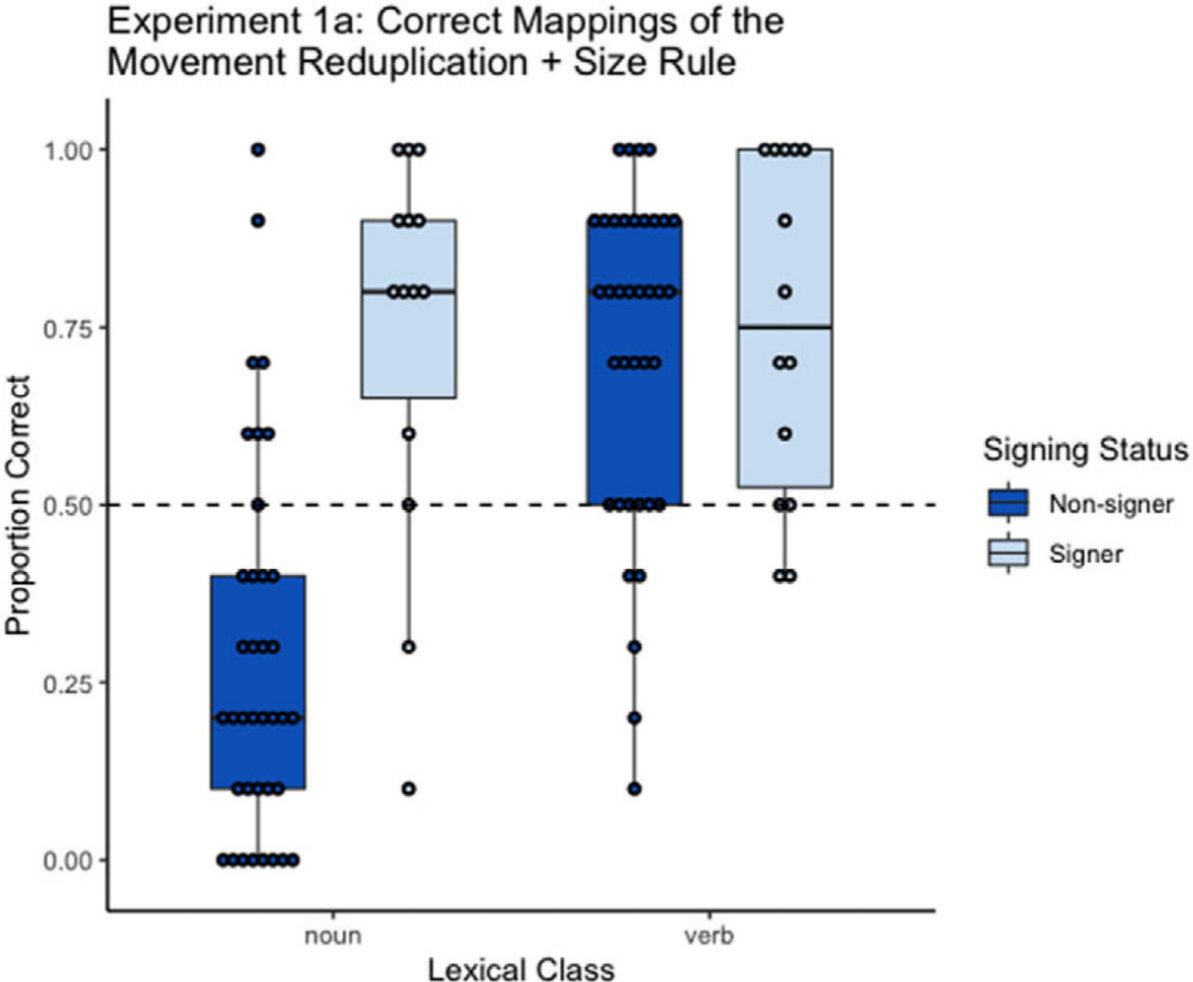
Proportion of correct matches made by non-signers and signers with the *movement reduplication* + *size* rule tested using the match-to-sample paradigm. Dots represent an individual participant’s mean. Whiskers represent the maximum and minimum scores except for extreme values. Dots outside of the whiskers are outliers. Dashed line indicates chance performance. Figure created using the ggplot2 package (Wickham, 2016) in the R programing environment.

We ran a mixed-effects logistic regression to examine participants’ success in matching object pictures with noun signs and action pictures with verb signs. We included item and participants as random intercepts, and lexical class, signing status, and the interaction between lexical class and signing status as fixed effects. All fixed factors were sum coded (lexical class: noun = −1, verb = +1; signing status: nonsigner = −1, signer = +1). We observed significant main effects of lexical class and signing status, and a significant interaction between signing status and lexical class (Table 1 and Fig. 3). The pattern of the data illustrates that nonsigners correctly matched verbs to action pictures, but not nouns with object pictures. Thus they did not use differences in movement to distinguish actions from objects. Instead, they treated most signs, regardless of movement reduplication, as actions – a bias that the match-to-sample design could not overcome. Signers, on the other hand, applied their knowledge of the ways that ASL distinguishes between nouns and verbs and correctly matched the signs to their corresponding meaning.

The results from the nonsigners could be explained in two ways. First, in the absence of a larger context, humans may be biased to treat any movement in a gesture as indicating an action. Previous work has documented this bias among nonsigners (Ortega et al., 2019). Second, the match-to-sample paradigm does not provide enough information for nonsigners to detect the relevant, albeit subtle contrasts in sign movement. Here, nonsigners saw either the noun or the verb variant, and had no opportunity to compare the movement information across the two signs. As such, for nonsigners the variations in movement were not necessarily contrastive. For signers fluent in ASL, this posed no challenge as they could draw on their knowledge of the systemic morphophonological contrasts in the language to answer correctly.

### 3.2. Experiment 1b: testing the sensitivity to the movement reduplication + size and movement size only rule in a between-subjects design with a forced-matching paradigm

To try to overcome the nonsigners’ bias to interpret all hand movements as actions and to provide participants with enough information to detect the relevant movement contrasts, we created a forced matching paradigm where participants saw both the noun and verb signs in each trial. Participants were asked to match each sign video with either a line drawing of an object or a line drawing representing the action associated with that object (Fig. 2b). Participants could not proceed to the next trial without matching both pictures to both signs.

#### 3.2.1. Method

##### Participants.

All 80 recruited MTurk workers completed all trials and passed all checks. The final sample included 52 males, 27 females, and 1 undisclosed (61 White, 7 Asian, 4 Hispanic or Latino, 1 Middle Eastern, 3 mixed race, and 4 undisclosed). Half of the participants viewed signs that differed according to the *movement reduplication* + *size* rule, and half of the participants viewed signs that differed according to the *movement size only* rule. We collected data for each condition separately, running the *movement reduplication* + *size* rule first, then recruiting new participants for the *movement size only* rule condition.

To confirm that deaf signers were sensitive to the *movement size only* rule, we tested deaf ASL signers in this condition alone. Nine of the deaf ASL signers that participated in Experiment 1a also completed Experiment 2 (males = 3, females = 6; 8 White, 1 Asian).

##### Design and procedure.

Using Qualtrics, we presented both the noun and verb signs on the screen. The location of the sign videos on the screen was randomized across trials. They could play each video as often as they wanted. Participants also saw line-drawings of the object and action referents related to the signs. Location of the images was randomized across trials. Participants were asked to match the videos with their corresponding images by clicking on one drawing, then dragging it to the location under the video to which it best corresponded. They had to match one picture with one video and the other picture with the second video in order to advance to the next trial. It took participants an average of 7 minutes to complete this study.

#### 3.2.2. Results and discussion

**Fig. 4.**
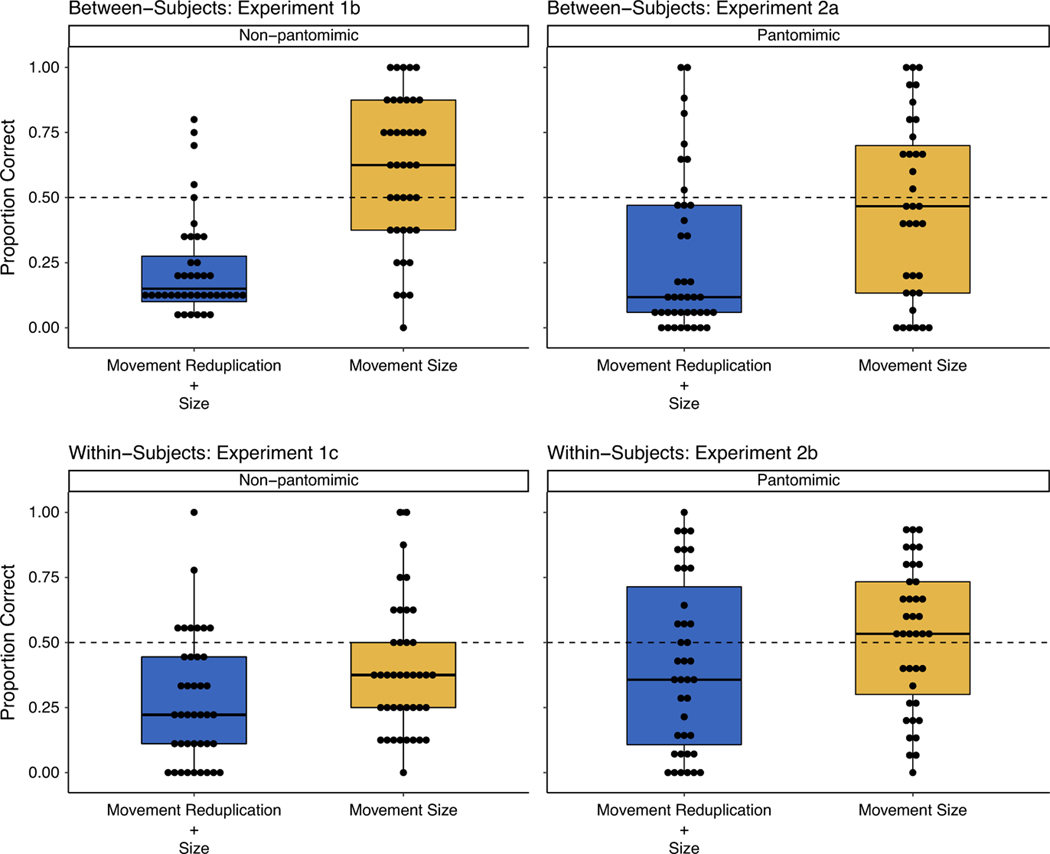
Proportion of correct matches made by non-signers across the forced matching paradigms that assessed sensitivity to each rule, both between- and within-subjects, as a function of whether the signs included non-pantomimic or pantomimic elements across all experiments. Dots represent an individual participant’s mean. Whiskers represent the maximum and minimum scores except for extreme values. Dots outside of the whiskers are outliers. Dashed line indicates chance performance. Figure created using the ggplot2 package (Wickham, 2016) in the R programing environment.

After data collection, we identified that one pantomimic sign (BATH) had been erroneously included among the exclusively nonpantomimic items. We removed the item from our analysis, although the pattern of findings remained qualitatively the same. We ran mixed-effects logistic regressions predicting accuracy in the forced-matching paradigm using the outlined model (see Section 2.3). We found that while nonsigners were significantly better at making correct mappings in the *movement size only* condition than in the *movement reduplication* + *size* condition, the intercept of the model indicates that their overall performance was significantly below chance. Thus, nonsigners did not systematically interpret these noun–verb contrasts in a sign-like way (see Fig. 4 and Table 2). Visual inspection of Fig. 4 illustrates two patterns. First, nonsigners systematically make the incorrect mapping, overwhelmingly preferring to map reduplicated movements to actions and nonreduplicated movements to objects. Second, there seems to be a trend for nonsigners to be more ASL-like in the *movement size only* condition.

Because the deaf ASL signers only completed one condition, we compared their performance to chance, finding that they correctly mapped ASL verbs to images of actions and ASL nouns to images of objects, and did so at very high rates (*M* = .89, *SD* = .17, *t*(8) = 6.84, *p* < 0.001, 95%CI[0.76–1.02]).

### 3.3. Experiment 1c: testing the sensitivity to the movement reduplication + size and movement size only rule in a within-subjects design with a forced-matching paradigm

In Experiment 1b we looked at the *movement reduplication* + *size* rule and *movement size only* rule separately with nonpantomimic signs. We demonstrated that non-signers showed some sensitivity to the *movement size only* rule, but were biased to treat signs with both contrastive reduplication and reduction as representing actions instead of objects. Because these two derivational strategies coexist in a sign language, we presented participants in Experiment 1c with a mix of nonpantomimic noun–verb pairs, half of which differed as a function of movement reduplication and reduction and half that different only in the reduction of movement size. Here we speculated that nonsigners may find the presentation of both rules confusing because reduplication is contrastive only in one of the pairings, but is present in a noncontrastive way in the second pairing.

#### 3.3.1. Method

##### Participants.

All 40 recruited MTurk workers completed all trials and passed all checks. The sample included 24 males and 16 females (31 White, 4 Asian, 1 Hispanic or Latino, 1 Black or African American, and 3 Mixed).

##### Design and procedure.

We used the forced-matching procedure outlined in Experiments 1b but added the within-subjects variable of movement rule to explicitly examine how participants interpreted the *movement reduplication* + *size* rule and the *movement size only* rule when the rules were presented together. We included the nine noun–verb pairs that followed the *movement size only rule*, and we randomly selected 9 of the 20 noun–verb pairs that followed the *movement reduplication* + *size* rule from the between-subjects experiment (see Appendix). The order of item presentation was randomly presented across participants. It took participants on average 11 minutes to complete the study.

#### 3.3.2. Results and discussion

We removed the erroneously included pantomimic sign BATH and then ran mixed-effects logistic regressions using the model outlined in Section 2.3. We observed a simple effect of rule that did not quite reach significance, with nonsigners making more correct matches with the *movement size only* rule than with the *movement reduplication* + *size* rule (Table 2 and Fig. 4). Participants’ overall performance, however, was still below chance.

When nonpantomimic noun–verb pairs that varied according to either rule were presented together, nonsigners continued to map small reduplicated movements to objects and large nonreduplicated movements to actions at below chance rates, performing in a similar way as when they saw the rule on its own (Experiment 1b). In the presentation of this set of signs, all nouns had reduplication and verbs varied with respect to reduplication. With either rule, movement size should be contrastive enough to help distinguish verbs from nouns. However, the bias to see reduplicated movements as actions may be so strong that nonsigners preferentially attended to differences in reduplication rather than differences in movement size and generalized that preference even when it was an irrelevant contrast.

## 4. Experiment 2: pantomimic signs

As noted above, signs with pantomimic iconicity where the hand represents a hand are preferentially associated with actions (Ortega & Özyürek, 2019; Padden et al., 2015; van Nispen et al., 2017) and are seen as more iconic by nonsigners (Sehyr & Emmorey, 2019). As such, pantomimic iconicity might override any existing biases to interpret some types of sign movements as associated with actions and others with objects. For example, the bias to treat pantomimic signs as actions may lead non-signers to ignore relevant movement differences that contrast nouns and verbs. Parallel to Experiment 1, we explored nonsigners’ sensitivity to the *movement reduplication* + *size* and the *movement size only* rules in a between-subjects design (Experiments 2a) and in a within-subjects design (Experiment 2b), when signs had pantomimic elements.

### 4.1. Experiment 2a: testing the sensitivity to the movement reduplication + size and movement size only rule in a between-subjects design with a forced-matching paradigm

#### 4.1.1. Method

##### Participants.

A total of 81 MTurk workers were recruited for this study. Forty-one MTurk workers completed all trials and passed all checks for the *movement size* + *reduplication* condition; we had one participant over the target sample size as a result of a fault in the MTurk system that allowed the last two participants to start the task simultaneously. For the *movement size only* condition, 35 of the 40 recruited MTurk workers completed all trials and passed all checks. The final sample across conditions included 43 males and 32 females, and 1 undisclosed (50 White, 17 Asian, 2 Hispanic or Latino, 5 Black or African American, and 2 mixed).

##### Design and procedure.

We used the procedure outlined in Experiment 1b but included 18 pantomimic noun–verb pairs that used the *movement reduplication* + *size* rule and 15 noun–verb pairs that had some pantomimic iconicity and used the *movement size only* rule (see Appendix). Presentation of items was randomized across participants. Participants took an average of 8 minutes to complete the study.

#### 4.1.2. Results and discussion

After data collection had been completed, we identified that a noun–verb pair that had been designated as nonpantomimic (PUT_ON_BRACELET/BRACELET) was erroneously included in the set of pantomimic items administered in this task. We removed that item for the reported analysis, although the results remained qualitatively the same as when it was included.

We ran mixed-effects logistic regressions following the model outlined in Section 2.3. We observed a significant effect of rule type with participants in the *movement size only* condition outperforming those in the *movement reduplication* + *size* condition (Table 2 and Fig. 4). Participants systematically mapped small reduplicated movements to actions and large nonreduplicated movements to objects, mirroring the bias that we observed in Experiment 1. In addition, nonsigners showed a modest bias toward interpreting a reduction in movement size in nonpantomimic signs as indicating objects, and larger movements as illustrating actions, when reduplicated movement was constant across nouns and verbs. Nevertheless, participants’ overall performance was below chance. Pantomimic iconicity did nothing to shift this bias.

### 4.2. Experiment 2b: testing the sensitivity to the movement reduplication + size and movement size only rule in a within-subjects design with a forced-matching paradigm

#### 4.2.1. Method

##### Participants.

Thirty-nine of the 40 recruited MTurk workers completed all trials and passed all checks. The final sample included 22 males and 17 females (24 Caucasian, 4 Asian, 4 Black or African American, 3 Hispanic or Latino, 1 Native American, 2 mixed, and 1 undisclosed).

##### Design and procedure.

We used the design and procedure outlined in Experiment 1c. To explicitly contrast the two movement rules, we used the 15 items from the movement reduplication + size condition and a randomly selected subset of 15 of the 18 movement size only condition of Experiment 2a (Table 1). Order of item presentation was randomized across participants. It took participants on average 11 minutes to complete the study.

#### 4.2.2. Results and discussion

We removed the erroneously included nonpantomimic noun–verb pair that used the *movement reduplication* + *size* rule (PUT_ON_BRACELET/BRACELET) from the analyses, although the results were qualitatively the same when both items were included in the analyses. We ran mixed-effects logistic regressions following the models outlined in Section 2.3. Participants performed better in the *movement size only* condition than in the *movement reduplication* + *size* condition (Table 2 and Fig. 4). Yet with pantomimic signs, nonsigners were nonsignificantly below chance in making correct matches across both conditions.

Visual inspection of Fig. 4 shows that the findings differ slightly from with those of the parallel experiment with nonpantomimic signs (Experiment 1c), where we observed a systematic bias toward mapping small reduced movements with actions and large movements, reduplicated or not, with objects – a pattern that is the opposite of what we see in sign languages. It is possible that an action bias associated with pantomimic iconicity competed with a bias to treat reduplication as an action. Yet, we observed no pantomimic action bias in the between-subjects design (Experiments 2a). The presentation of both rules together in within-subjects may have reduced the bias seen in the between-subjects experiment.

## 5. Item analyses

**Fig. 5.**
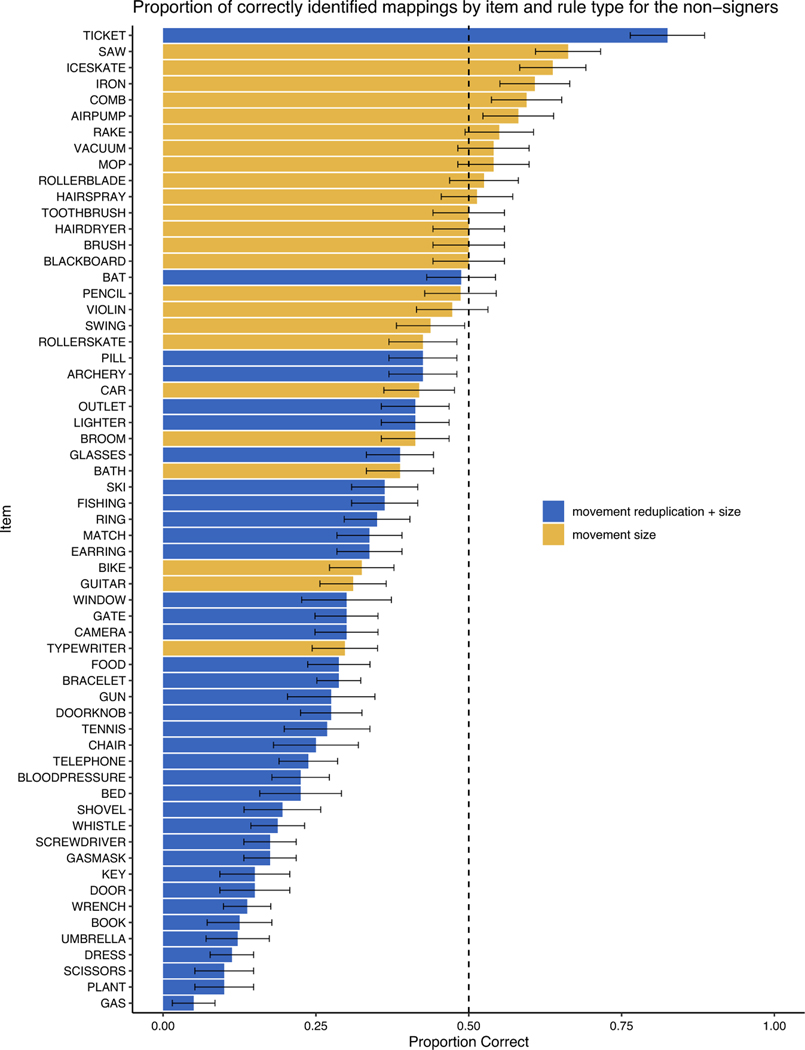
Proportion correct for each item collapsed across all experiments. The dotted line represents chance performance. The error bars represent standard error (SE). Figure created using the ggplot2 package (Wickham, 2016) in the R programing environment.

To better understand the effect of item, we combined the data from across all of the experiments and plotted accuracy for each item in Fig. 5. In addition, for each item, we compared performance to chance using an exact binomal test applying a Bonferroni correction for multiple comparisons, setting the *p*-value as 0.0008. Performance on all of the items was at or significantly below chance in their mapping of the movement patterns to nouns and verbs (see Supplementary Table S1). What is clear from Fig. 5 is that for items where reduplication is contrastive, nonsigners were less likely to make an ASL-like mapping, preferring to treat reduplicated movement as “action-like” instead of “object-like.”

## 6. Investigating the effect of pantomimic iconicity

Our plan from the outset was to investigate nonpantomimic and pantomimic iconicity separately. Visual inspection of the data presented in Fig. 4 does not indicate that pantomimic iconicity improved participants’ ability to make correct matches. But to explicitly test this possibility we combined the data from all of the experiments and ran a mixed effects logistic regression (see Section 2.3). We continued to observe the main effect of rule type (see Table 3) and no effect of iconicity type. There was no interaction between rule type and iconicity type – the effect of rule was the same for both types of iconicity. Thus, participants’ choices were systematically less ASL-like when the relevant contrast involved *movement reduplication* + *size* than with *movement size* alone and pantomimic iconicity did nothing to shift this pattern of performance.

## 7. General discussion

Across a series of experiments, we investigated whether morphophonological distinctions between nouns and verbs in ASL are iconically driven by the way humans construe objects and actions. We found some evidence that nonsigners were more accurate in mapping small movements to objects and large movements to actions than in mapping small reduplicated movements to objects and large nonreduplicated movements to actions. Nevertheless, their overall mapping accuracy was either at or below chance indicating that contrary to our original prediction, they did not automatically make the same mappings observed in ASL.

Nonsigners’ performance exhibited a set of biases that deviated from the patterns observed in sign languages. First, in the match-to-sample paradigm (Experiment 1a) where participants were presented with a single sign, nonsigners treated the majority of signs as verbs, reflecting a bias to treat any human movement as verb-like. People typically construe human movements as depicting events, and they attend to small changes in movement properties such as acceleration and velocity to reliably identify boundaries between events (e.g., Zacks et al., 2009). Ortega et al. (2019) also found that nonsigners showed a strong bias to assume that signs from Sign Language of the Netherlands (NGT) refer to actions and not objects. A similar bias has been seen in nonsigners’ creation of novel gestures about objects; when nonsigners are asked to silently gesture about objects, they prefer to depict the action affordances of an object (e.g., how a toothbrush is handled) instead of its physical features (e.g., the size and shape of a toothbrush; Ortega & Özyürek, 2019; van Nispen et al., 2017).

Second, when we made the morphophonological contrasts more salient by presenting both nouns and verbs simultaneously, nonsigners systematically interpreted movement reduplication as more action-like and nonreduplicated movement as more object-like (Fig. 4), displaying a sensitivity to the movement reduplication and size contrast between nouns and verbs. Their interpretation of this contrast, however, is the opposite of how reduplication is used to derive nouns from verbs across many sign languages. Thus, the bias that nonsigners have in interpreting reduplicated movement is likely not the source of the morphophonological distinction used to distinguish nouns from verbs found in mature sign languages.

The analysis consistently revealed more ASL-like mappings for items that followed the *movement size only* rule than for the *movement reduplication* + *size* rule. However, this improvement in performance rarely rose to above chance levels. We suggest that the significant difference between conditions is a result of nonsigners’ systematic below chance performance in the *movement reduplication* + *size* condition where they regularly interpreted reduplication as action-like, and not as a result of treating small movements as more object-like (e.g., Wilcox, 2004). Nevertheless, at least one other study has shown that nonsigners slightly prefer to treat gestures with long, continuous movement as more action-like and those with small constrained movements as more object-like (Verhoef & Lepic, 2020). Thus, whether nonsigners treat movement size as a meaningful cue to lexical category merits further exploration.

We predicted that when the two rules were presented simultaneously, as in Experiments 1c and 2b, any sensitivity to the mappings that nonsigners showed when the rules were presented on their own might disappear because it would be difficult to extract the relevant contrasts. Visual inspection of the results does not yield a straightforward account. With nonpantomimic signs, nonsigners continued to interpret reduplicated movements as actions and nonreduplicated movements as objects, but their performance in the *movement size only* condition was now less accurate. With pantomimic signs, nonsigners no longer systematically interpreted reduplication as verb-like, and their performance with both rule types was at chance. Regardless, nonsigners’ choices did not parallel the linguistic mappings associated with either rule in sign languages.

Given the robust evidence that nonsigners treat pantomimic iconicity as more action-like, we speculated that we might see improved rule mapping with pantomimic signs. Pantomimic iconicity, however, did not affect the biases that we observed with nonpantomimic signs. One difference between our study and other studies that have previously demonstrated a strong association between pantomimic iconicity and action concepts is that our nouns and verbs differed as a function of movement and not handshape. The preferential association of pantomimic iconicity with actions may be limited to cases where handshape is a contrastive feature distinguishing nouns and verbs (Ortega & Özyürek, 2019; Padden et al., 2015; Verhoef et al., 2018).

Investigation of sign languages at their earliest stage of emergence can help us understand whether sign languages’ morphophonological distinctions used to differentiate nouns and verbs might arise from a widespread iconic bias among humans to see large articulated movements as actions and small reduplicated movements as deemphasizing actions and inviting an object interpretation of signs (Lepic & Padden, 2017; Wilcox, 2004). Homesigners, deaf children who create their own family sign communication, seem to show some evidence of the movement size bias. They tend to produce larger sign movements using more proximal joints (e.g., shoulders and elbows) to communicate about actions, and smaller sign movements when they communicate about objects (Abner et al., 2019; Goldin-Meadow et al., 1994). Emerging sign languages, such as the village sign language Al-Sayyid Bedouin Sign Language (ABSL) and the community sign language Nicaraguan Sign Language (NSL), also show an early emergence of large sign movements to express actions, although NSL seems to exhibit this pattern more reliably than ABSL (Abner et al., 2019; Tkachman & Sandler, 2013). Our observation that in at least one case (Experiment 1b), nonsigners more systematically interpreted large movements as actions for nonpantomimic signs lends some support for an iconic bias for the *movement size only* morphophonological distinction early in language emergence; however, this bias is by no means robustly present at the inception of a sign language.

The use of the *movement reduplication* + *size* distinction is far less prevalent than the *movement size only* distinction in homesign and emerging sign languages. In a study of a single homesigner in the United States, Goldin-Meadow et al. (1994) found that gestures for objects tended to be “abbreviated” relative to those used for actions, and the nature of this abbreviation was, in part, the elimination of reduplicated or “bidirectional” movement (pp. 283–284). That is, the homesigner exhibited the same bias nonsigners exhibited, associating reduplicated movement with actions. In a recent study of four homesigners in Nicaragua, three homesigners used reduplication in a contrastive way to distinguish objects from actions, but they did so only for iterable actions (Abner et al., 2019). Only with later cohorts of NSL signers do we see a more systematic use of reduplication to mark nouns across both iterable and noniterable events (Abner et al., 2019). Thus, an initial bias to treat reduplication as action-like may make it more difficult for a sign language to immediately adopt reduplication as a strategy for noun derivation.

There are several possible reasons for the more gradual adoption of these morphophonological distinctions in sign languages. First, these distinctions are not readily available to nonsigners who have a bias to interpret reduplication as action-like instead of object-like. Second, handshape may be a more salient cue than movement to distinguish objects from actions for nonsigners and homesigners during language creation (Hunsicker & Goldin-Meadow, 2013; Padden et al., 2015; Verhoef et al., 2018). Finally, as a language emerges, other linguistic pressures may shift the language away from any initial bias. For example, many different sign languages leverage space and movement for verbal morphology, marking agreement, number, and aspect (see Sandler & Lillo-Martin, 2006 for a review). Relying on movement reduplication to mark actions could constrain the ways a signer can use space and directional movement to mark other grammatical functions. Interestingly, the use of space for grammatical features emerges over time (Kocab et al., 2015; Pyers et al., 2010; Pyers & Senghas, 2007; Senghas, 2003; Senghas & Coppola, 2001), and there may be an inverse relationship between the emergence of spatial grammar and the use of reduplication to represent actions, although we need additional research to explore this possibility. Relatedly, the use of movement reduplication to mark objects may arise alongside a phonological shift restricting the size of the signing movements associated with nouns; some preliminary evidence shows that nouns in ASL are articulated in a smaller signing space compared to verbs (Sehyr et al., 2019), and reduplication may increase the perceptual salience of signs articulated with smaller movements.

The robustness of various morphophonological distinctions between noun–verb pairs in sign languages is certainly debatable. While elicited production (Abner et al., 2019) and assessed comprehension (Experiments 1a and 1b) of these noun–verb pairs in ASL elicits reliable distinctions from fluent signers, the occurrence of these distinctions may be less robust in connected discourse or naturalistic conversation (Bouchard et al., 2005; Johnson, 2001; Voghel, 2005). Signers instead may favor other features like word order, spatial grammar, and context over morphophonology to communicate and interpret the meaning of a potential noun or verb form (Voghel, 2005). One intriguing possibility is that linguistic competition with human cognitive construals may lead to less consistent use of those linguistic features in naturalistic conversation, although we need more research to explore this possibility.

## 8. Conclusion

When building a new language, language creators can draw from an array of resources including iconicity. Crucially, the available iconic mappings are shaped by how humans construe their world. In the case of distinguishing objects and actions, humans come to the table with a strong bias to associate movement and reduplication with actions rather than with objects, a bias that reflects an iconic mapping between motion and action events that is absent for static entities. The availability of an initial iconic mapping at the inception of a language, however, does not ensure its survival. As a language builds its phonological, morphological, syntactic, and discursive systems, any initial mapping may be reshaped or even overridden to align with the constraints of the system. Moreover, the way humans interpret iconic mappings is shaped by their experiences, including sign language experience (Occhino et al., 2017; Sehyr & Emmorey, 2019). The end result may nevertheless be iconic, reflecting a new way to construe objects and actions that has been shaped by the language system and the experience of the users. Thus, the nature of the structural mapping between a linguistic form and semantic features is dynamic and can change over time.

## Supplementary Material

1

## Figures and Tables

**Table 1.** Fixed and random effects predicting accuracy with the movement reduplication + size in the match to sample paradigm in Experiment 1a

	Experiment 1a: Match-to-sample, Movement reduplication + size		
	
Predictors	Odds ratios	CI	*p*

(Intercept)	0.36	0.25–0.52	**<0.001**
Lexical Class (verb)	7.44	5.27–10.50	**<0.001**
Hearing status (signer)	9.76	5.13–18.59	**<0.001**
Lexical Class (verb) × Hearing status (signer) Random effects	0.14	0.07–0.27	**<0.001**
*σ*^2^		3.29	
*τ*_00ResponseID_		0.47	
*τ*_00ITEM_		0.15	
ICC		0.16	
*N*_ResponseID_		51	
*N*_ITEM_		20	
Observations		1,020	
Marginal *R*^2^/Conditional *R*^2^		0.21/0.34	

*Note*. Table generated using the tab_model function from the SJPlot package version 2.6.2 for the R programming environment. Fixed factors were sum coded: (lexical class: noun = −1, verb = +1; signing status: nonsigner = −1, signer = +1) glmer(correct ~ Lexical_Class×Hearing_status+(1 | ResponseID)+(1 | ITEM), data=df, family =binomial, control=glmerControl(optimizer = “bobyqa”)).

**Table 2.** Fixed and random effects predicting accuracy Experiments 1b–2b

Test text	Experiment 1b: Nonpantomimic between-subjects^a^			Experiment 1c: Nonpantomimic within-subjects^b^			Experiment 2a: Pantomimic between-subjects^a^			Experiment 2b: Pantomimic within-subjects^b^		
				
Predictors	Odds ratios	CI	*P*	Odds ratios	CI	*P*	Odds ratios	CI	*P*	Odds ratios	CI	*P*

(Intercept)	0.17	0.09–0.32	**<0.001**	0.26	0.14–0.49	**<0.001**	0.16	0.07–0.36	**<0.001**	0.53	0.25–1.10	0.087
Rule type (Movement size)	10.20	3.73–27.90	**<0.001**	2.04	0.95–4.39	0.066	4.31	1.32–14.04	**0.015**	2.10	1.16–3.80	**0.014**
Random effects												
*σ*^2^		3.29			3.29			3.29			3.29	
*τ*_00_		1.69_ResponseID_			1.70_ResponseID_			5.37_ResponseID_			4.15_ResponseID_	
		0.91_ITEM_			0.33_ITEM_			0.31_ITEM_			0.24_ITEM_	
*τ*_11_					1.51_ResponseID.rule_sc1_						1.30_ResponseID.rule_sc1_	
*ρ*_01_					−0.70_ResponseID_						−0.81_ResponseID_	
ICC		0.44			0.34			0.63			0.49	
*N*		80_ResponseID_			40_ResponseID_			76_ResponseID_			39_ResponseID_	
		29_ITEM_			18_ITEM_			33_ITEM_			30_ITEM_	
Observations		1,160			720			1,263			1,170	
Marginal *R*^2^/Conditional *R*^2^		0.16/0.53			0.03/0.35			0.06/0.65			0.02/0.50	

*Note.* Table generated using the tab_model function from the SJPlot package version 2.6.2 for the R programming environment. Fixed factor of Rule Type was sum coded (movement reduplication + size = −1, movement size only = +1). Bolded p-values are statistically significant.

a glmer(correct ~ rule_type + (1 | ResponseID) + (1 | ITEM), data = df, family = binomial, control = glmerControl(optimizer = “bobyqa”)).

b glmer(correct ~ rule_type + (1 + rule_type|ResponseID) + (1 | ITEM), data = df, family = binomial, control = glmerControl(optimizer = “bobyqa”).

**Table 3.** Fixed and random effects predicting accuracy combining data from all experiments for nonsigners

	Correct mapping		
	
Predictors	Odds ratios	CI	*p*

(Intercept)	0.24	0.14–0.40	**<0.001**
Rule type [Movement size]	3.82	1.78–8.20	**0.001**
Iconicity type [Pantomimic]	1.21	0.60–2.41	0.593
Rule type [Movement size] × Iconicity type [Pantomimic] Random effects	0.69	0.26–1.83	0.452
*σ*^2^		3.29	
*τ*_00 ResponseID_		3.37	
*τ*_00 item_		0.53	
*τ*_11 ResponseID.rule_sc1_		1.61	
*ρ*_01 ResponseID_		−0.56	
ICC		0.51	
*N*_ResponseID_		235	
*N*_item_		61	
Observations		4,313	
Marginal *R*^2^ /Conditional *R*^2^		0.05/0.54	

*Note.* Table generated using the tab_model function from the SJPlot package version 2.6.2 for the R programming environment. glmer(correct ~ rule_type × Iconicity_type_sc + (1 + rule_sc| ResponseID) + (1 | item), data = df, hearing, family = binomial, control = glmerControl(optimizer =“bobyqa”)).

## Footnotes

1 This decision was based on a power analysis by G*Power (version 3.1.9.6), which indicated a sample of 37 in each condition was needed to yield an effect size of .55 with a power of .90. We oversampled, targeting 40 and 80 participants for the within- and between-subject conditions, respectively.

## APPENDIX

### Appendix

#### A. Appendix

**Table A.** ASL noun–verb pairs that appeared in each experiment

Rule	Nonpantomimic (VERB/NOUN)	Pantomimic (VERB/NOUN)

Movement reduplication + size	Experiment 1a and 1b GO_TO_BED/BED TAKE_BLOOD_PRESSURE/BLOOD_PRESSURE^a^ OPEN_BOOK/BOOK PUT_ON_BRACELET/BRACELET^a^ SIT/CHAIR GET_DRESSED/DRESS OPEN_DOOR/DOOR FILL_WITH_GAS/GAS OPEN_GATE/GATE^a^ SHOOT/GUN TURN_ON_IGNITION/KEY PLUG_IN/OUTLET^a^ GROW/PLANT CUT/SCISSORS TO_SKI/SKIS^a^ GIVE_A_TICKET/TICKET BLOW_WHISTLE/WHISTLE^a^ OPEN_WINDOW/WINDOW TURN_NUT/WRENCH^a^ SCREW_IN/SCREWDRIVER^a^	Experiment 2a SHOOT_ARROW/ARROW^b^ TO_BAT/BAT^b^ TO_PHOTOGRAPH/CAMERA^b^ TURN_DOORKNOB/DOORKNOB^b^ PUT_ON_EARING/EARING^b^ TO_FISH/FISHING^b^ EAT/FOOD^b^ PUT_ON_GAS_MASK/GAS_MASK^b^ PUT_ON_GLASSES/GLASSES^b^ FLICK_ON_LIGHTER/ LIGHTER^b^ LIGHT_MATCH/MATCH^b^ TAKE_PILL/PILL^b^ PUT_RING_ON/RING^b^ TO_SHOVEL/SHOVEL^b^ TO_TELEPHONE/TELEPHONE SWING_RACKET/TENNIS OPEN_UMBRELLA/UMBRELLA
Movement size	Experiment 1b^a^ RIDE_BIKE/BICYCLE SWEEP/BROOM TO_ICESKATE/ICESKATES TO_RAKE/RAKE TO_ROLLERBLADE/ROLLERBLADES TO_ROLLERSKATE/ROLLERSKATES TO_SAW/SAW TO_SWING/SWING	Experiment 2b^b^ PUMP_AIR/AIR_PUMP TO_ERASE/ERASER TO_BRUSH/BRUSH DRIVE/CAR TO_COMB/COMB STRUM/GUITAR TO_BLOWDRY/BLOWDRYER TO_HAIRSPRAY/HAIRSPRAY TO_IRON/IRON TO_MOP/MOP TO_WRITE/PENCIL TO_BRUSH_TEETH/TOOTHBRUSH TO_TYPE/TYPEWRITER TO_VACUUM/VACUUM_CLEANER PLAY_VIOLIN/VIOLIN

a Also appeared in Experiment 1c.

b Also appeared in Experiment 2b.
