# Supplementary material for: The iconic motivation for the morphophonological distinction between noun–verb pairs in American Sign Language does not reflect common human construals of objects and actions
Source: Lang Cogn. Author manuscript; Available in PMC 2022 Dec 1. (PMC9681175; doi:10.1017/langcog.2022.20)
Supplement: 1 [file NIHMS1830289-supplement-1.docx]

Table S1. Table of Item Results from the Exact Binomial Tests Across all Experiments

|  |  |  |  |  |  | 95% Confidence Interval | |
| --- | --- | --- | --- | --- | --- | --- | --- |
| Item | Total Possible | Correct Identification | Estimate | Statistic | *P^a^* | Lower Bound | Upper Bound |
| AIRPUMP | 74 | 43 | 0.419 | 31 | 0.201 | 0.305 | 0.539 |
| ARCHERY | 80 | 34 | 0.575 | 46 | 0.219 | 0.459 | 0.685 |
| BAT | 80 | 39 | 0.513 | 41 | 0.911 | 0.398 | 0.626 |
| BATH | 80 | 31 | 0.613 | 49 | 0.057 | 0.497 | 0.719 |
| BED | 60 | 10 | 0.833 | 50 | 0 | 0.715 | 0.917 |
| BIKE | 80 | 26 | 0.675 | 54 | 0.002 | 0.561 | 0.776 |
| BLACKBOARD | 74 | 37 | 0.5 | 37 | 1 | 0.381 | 0.619 |
| BLOODPRESSURE | 100 | 21 | 0.79 | 79 | 0 | 0.697 | 0.865 |
| BOOK | 60 | 12 | 0.8 | 48 | 0 | 0.677 | 0.892 |
| BRACELET | 177 | 56 | 0.684 | 121 | 0 | 0.61 | 0.751 |
| BROOM | 80 | 33 | 0.588 | 47 | 0.146 | 0.472 | 0.696 |
| BRUSH | 74 | 37 | 0.5 | 37 | 1 | 0.381 | 0.619 |
| CAMERA | 80 | 24 | 0.7 | 56 | 0 | 0.587 | 0.797 |
| CAR | 74 | 31 | 0.581 | 43 | 0.201 | 0.461 | 0.695 |
| CHAIR | 57 | 15 | 0.737 | 42 | 0 | 0.603 | 0.845 |
| COMB | 74 | 44 | 0.405 | 30 | 0.13 | 0.293 | 0.526 |
| DOOR | 60 | 11 | 0.817 | 49 | 0 | 0.696 | 0.905 |
| DOORKNOB | 80 | 22 | 0.725 | 58 | 0 | 0.614 | 0.819 |
| DRESS | 100 | 16 | 0.84 | 84 | 0 | 0.753 | 0.906 |
| EARRING | 80 | 27 | 0.663 | 53 | 0.005 | 0.548 | 0.764 |
| FISHING | 80 | 29 | 0.638 | 51 | 0.018 | 0.522 | 0.742 |
| FOOD | 80 | 23 | 0.713 | 57 | 0 | 0.6 | 0.808 |
| GAS | 57 | 4 | 0.93 | 53 | 0 | 0.83 | 0.981 |
| GASMASK | 80 | 14 | 0.825 | 66 | 0 | 0.724 | 0.901 |
| GATE | 97 | 29 | 0.701 | 68 | 0 | 0.6 | 0.79 |
| GLASSES | 80 | 31 | 0.613 | 49 | 0.057 | 0.497 | 0.719 |
| GUITAR | 74 | 23 | 0.689 | 51 | 0.002 | 0.571 | 0.792 |
| GUN | 57 | 21 | 0.632 | 36 | 0.063 | 0.493 | 0.756 |
| HAIRDRYER | 74 | 37 | 0.5 | 37 | 1 | 0.381 | 0.619 |
| HAIRSPRAY | 74 | 38 | 0.486 | 36 | 0.908 | 0.369 | 0.606 |
| ICESKATE | 80 | 51 | 0.363 | 29 | 0.018 | 0.258 | 0.478 |
| IRON | 74 | 45 | 0.392 | 29 | 0.081 | 0.28 | 0.512 |
| KEY | 60 | 7 | 0.883 | 53 | 0 | 0.774 | 0.952 |
| LIGHTER | 80 | 33 | 0.588 | 47 | 0.146 | 0.472 | 0.696 |
| MATCH | 80 | 27 | 0.663 | 53 | 0.005 | 0.548 | 0.764 |
| MOP | 74 | 40 | 0.459 | 34 | 0.561 | 0.343 | 0.579 |
| OUTLET | 100 | 37 | 0.63 | 63 | 0.012 | 0.528 | 0.724 |
| PENCIL | 74 | 36 | 0.514 | 38 | 0.908 | 0.394 | 0.631 |
| PILL | 80 | 34 | 0.575 | 46 | 0.219 | 0.459 | 0.685 |
| PLANT | 60 | 9 | 0.85 | 51 | 0 | 0.734 | 0.929 |
| RAKE | 80 | 44 | 0.45 | 36 | 0.434 | 0.338 | 0.565 |
| RING | 80 | 28 | 0.65 | 52 | 0.01 | 0.535 | 0.753 |
| ROLLERBLADE | 80 | 42 | 0.475 | 38 | 0.738 | 0.362 | 0.59 |
| ROLLERSKATE | 80 | 34 | 0.575 | 46 | 0.219 | 0.459 | 0.685 |
| SAW | 80 | 53 | 0.338 | 27 | 0.005 | 0.236 | 0.452 |
| SCISSORS | 60 | 12 | 0.8 | 48 | 0 | 0.677 | 0.892 |
| SCREWDRIVER | 97 | 18 | 0.814 | 79 | 0 | 0.723 | 0.886 |
| SHOVEL | 41 | 8 | 0.805 | 33 | 0 | 0.651 | 0.912 |
| SKI | 97 | 39 | 0.598 | 58 | 0.067 | 0.493 | 0.696 |
| SWING | 80 | 35 | 0.563 | 45 | 0.314 | 0.447 | 0.673 |
| TELEPHONE | 80 | 19 | 0.763 | 61 | 0 | 0.654 | 0.851 |
| TENNIS | 41 | 11 | 0.732 | 30 | 0.004 | 0.571 | 0.858 |
| TICKET | 57 | 38 | 0.333 | 19 | 0.016 | 0.214 | 0.471 |
| TOOTHBRUSH | 74 | 37 | 0.5 | 37 | 1 | 0.381 | 0.619 |
| TYPEWRITER | 74 | 22 | 0.703 | 52 | 0.001 | 0.585 | 0.803 |
| UMBRELLA | 41 | 5 | 0.878 | 36 | 0 | 0.738 | 0.959 |
| VACUUM | 74 | 40 | 0.459 | 34 | 0.561 | 0.343 | 0.579 |
| VIOLIN | 74 | 35 | 0.527 | 39 | 0.728 | 0.407 | 0.644 |
| WHISTLE | 97 | 21 | 0.784 | 76 | 0 | 0.688 | 0.861 |
| WINDOW | 57 | 18 | 0.684 | 39 | 0.008 | 0.548 | 0.801 |
| WRENCH | 100 | 12 | 0.88 | 88 | 0 | 0.8 | 0.936 |

^a^ *significance set at p<.0008 using a Bonferroni correction*
